# The origin of black and white coloration of the Asian tiger mosquito *Aedes albopictus* (Diptera: Culicidae)

**DOI:** 10.3762/bjnano.14.41

**Published:** 2023-04-17

**Authors:** Manuela Rebora, Gianandrea Salerno, Silvana Piersanti, Alexander Kovalev, Stanislav N Gorb

**Affiliations:** 1 Dipartimento di Chimica, Biologia e Biotecnologie, University of Perugia, Via Elce di Sotto 8, 06121 Perugia, Italy; 2 Dipartimento di Scienze Agrarie, Alimentari e Ambientali, University of Perugia, Borgo XX Giugno, 06121 Perugia, Italy; 3 Department of Functional Morphology and Biomechanics, Zoological Institute, Kiel University, Am Botanischen Garten 9, 24098 Kiel, Germany

**Keywords:** biological optics, insects, nanostructure, scales, structural white

## Abstract

Micro- and nanostructures of the white and black scales on the tarsi of the mosquito *Aedes albopictus* are analysed using scanning electron microscopy, transmission electron microscopy, and fluorescence microscopy. Reflectance spectra of the white areas are measured. No clear difference is present in the morphology of micro- and nanostructures of black and white scales in SEM and TEM, but black scales contain a dark pigment. The white colour of the scales has a structural origin. The structural white produced by the micro- and nanostructures of the scales on the tarsi of *Ae. albopictus* appears bright and is angle-dependent, since the reflected light changes according to the angle detection and according to the tarsus orientation. The optical appearance of the scale system of *Ae. albopictus* has a complex nature and can be explained by the combination of several effects. Among them, multiple refraction and reflection on the micro- and nanostructures of the scales are mainly responsible for the white appearance. The results suggest that mosquito scales, in addition to their superhydrophobic function, produce structural white. The biological role of white and black patches in mate recognition and defensive behaviour in the mosquitoes of the genus *Aedes* is hypothesized.

## Introduction

Body color (coloration) and light signals (bioluminescence) have a fundamental role in insect inter- and intra-specific visual communication allowing for species recognition, mating, prey capture, and predator avoidance [1]. Insect colours may be due to pigments (e.g., melanins, carotenoids, ommochromes, and pteridins situated in the cuticle or under a transparent cuticle) [2] able to absorb visible electromagnetic radiation in a selective way, or due to various physical phenomena, such as reflection, refraction, interference, diffraction and scattering, that cause the selective reflection of light [3]. Quite often, structural colors are present together with pigment colors, to increase or to reduce the brightness and to produce particular effects [4]. Insect exoskeletons with their multilayered internal organisation and the cuticular micro- and nanostructures present on the surface offer remarkable examples of structural colours in the animal kingdom. The relationship between shining (sometimes iridescent) colors and surface microstructures located on scales has been extensively studied in butterflies, especially in the *Morpho* genus [4–7].

Among Diptera, representatives from the family Culicidae bear scales typically covering their body [8–9]. Their shape and structure vary among different taxa and have been previously used as a taxonomic character [10]. The function of the body scales in mosquitoes is probably related to the superhydrophobicity of their body surface, as an adaptation of an aquatic insect to the subaerial life at the adult stage. Indeed, leg scales with their nanostructures are able to entrap air [11–13] and play an important role in contact with water during egg laying, giving the mosquito high water buoyancy and floating ability, and during emergence from the aquatic pupa, preventing adults from wetting and allowing them to fly away from the water surface without being trapped by capillary forces.

The scales on the mosquito body can also produce a colouration pattern, which is often used in the species determination [14–15]. Notwithstanding such scales are rather similar to those of butterfly wings [16], the mosquito scale nanostructures have not been deeply investigated so far regarding the structural colours they generate. Structural colours are common in insects [4] and have been described mainly in Lepidoptera and Coleoptera [16–17]. As far as Diptera are concerned, investigations on structural colours are scanty.

The aim of the present investigation is to describe in detail the nanostructures and microstructures of the scales in the Asian tiger mosquito *Aedes albopictus* Skuse (Diptera: Culicidae). The species belonging to this genus (among which *Ae. aegypti* L. is present) are visually distinctive, because they have noticeable black and white markings on their bodies and their legs, whose different distributions in different species allows for species identification. The ultrastructure of the white and black scales on the hindlegs of *Ae. albopictus* is analysed using scanning electron microscopy, transmission electron microscopy, and fluorescence microscopy. Moreover, reflectance spectra of the white areas are measured. The scales are present also on other body parts, but we mainly focused our attention on the hindlegs, because of their bright white stripes.

*Ae. albopictus* or Asian tiger mosquito is an invasive species native to tropical and subtropical areas of Southeast Asia, nowadays established in all Mediterranean countries. It is recognised as one of the 100 most invasive species in the world being an aggressive day-biting species. It is of high medical importance as a vector of chikungunya virus, dengue virus, and dirofilariasis [18]. Detailed studies regarding the optical properties of the body surface of insects, such as *Ae. albopictus* can be relevant to both species recognition and surveillance. Moreover, these studies increase our knowledge on inter- and intraspecific communication of these dangerous species, thus helping to develop new control methods.

## Results

**Figure 1:**
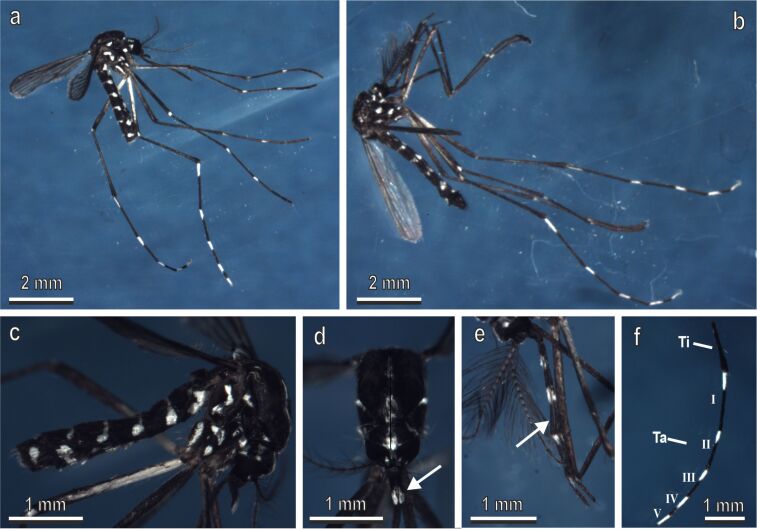
Adults of *Aedes albopictus* under a stereomicroscope. Female (a) and male (b) typically characterised by the presence of white and black areas on their body. (c) Detail of the abdomen and thorax of the female. (d) Detail of the head and maxillary palps (arrow) of a female. (e) Detail of the maxillary palp (arrow) of a male. (f) Detail of the tarsi (Ta) of the hindlegs of the female. Note that each segment is white in its proximal portion and black in its distal portion except the last segment which is totally white. Ti, tibia.

The body of the adult (male and female) of *Ae. albopictus* is typically characterised by the presence of white and black areas (Figure 1). White patches are present on abdominal segments, thorax, head, and legs of both sexes. They are particularly evident on the hindlegs (Figure 1c–f). Males and females differ in size and in shape of antenna and mouthpart, but their hindlegs are similar in colour and shape. Their tarsi are characterised by five segments, whose length decreases from the first to the last tarsal segment. Each segment is white in its proximal portion and black in its distal portion, except for the last segment, which is totally white (Figure 1f).

**Figure 2:**
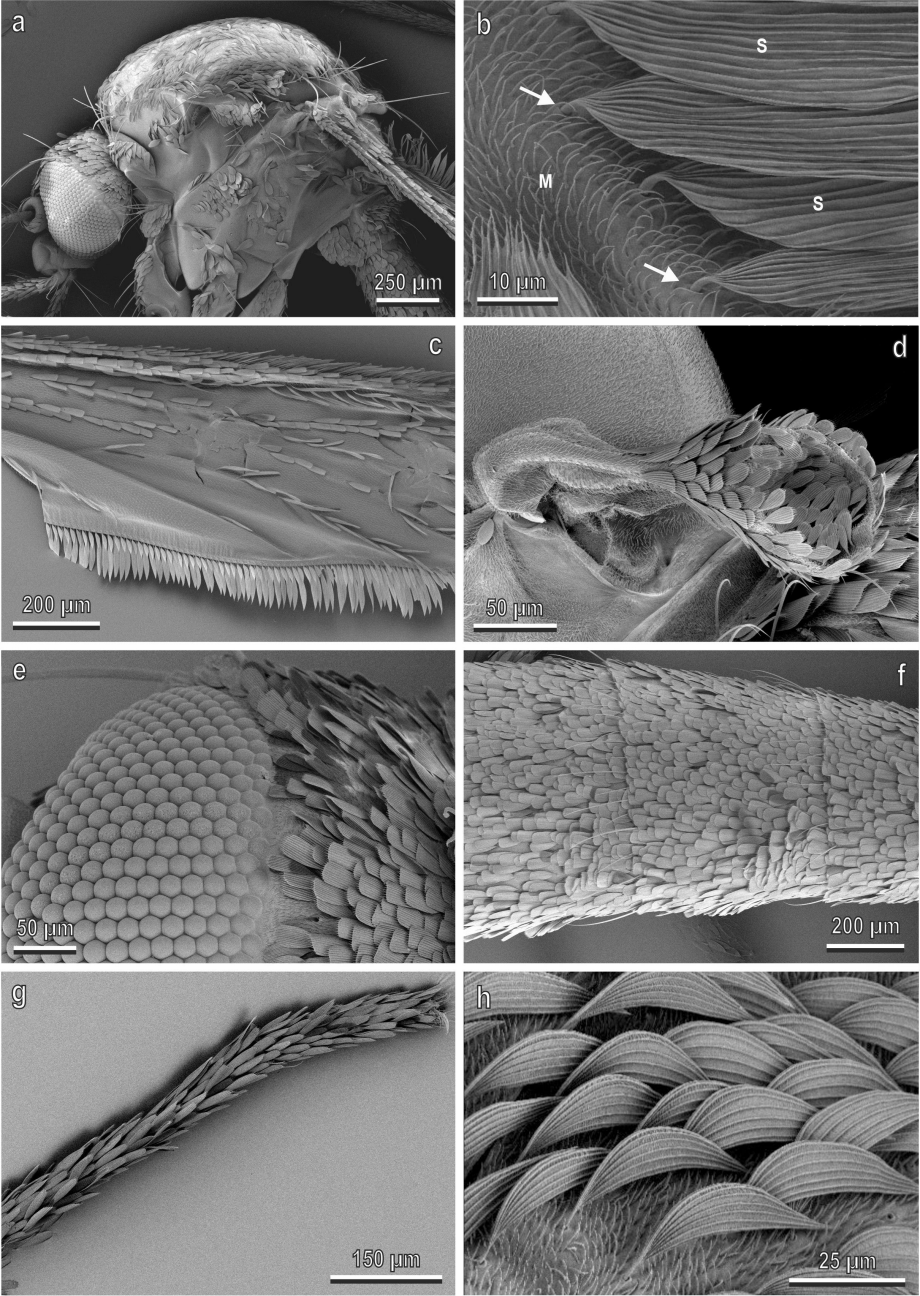
*Aedes albopictus* (female) in a cryo-SEM. (a) Lateral view of the thorax of *Ae. albopictus* covered with scales and microtrichia. (b) Detail of (a) showing microtrichia (M) and scales (S) with their articulated insertion (arrow) in the cuticle. (c–g) Scales with different shape located on the different body parts such as wings (c), halters (d), head (e), abdomen (f) and legs (g). (h) Detail of dorsal side of the thorax with falcate scales.

Observations with SEM reveal that the body of *Ae. albopictus* is covered with scales and microtrichia (Figure 2). Scales of different shape are present on different body parts. Spatulate scales are the most common kind of scales. They are found on the thorax (Figure 2a,b), wings (Figure 2c), halters (Figure 2d), head (Figure 2e), abdomen (Figure 2f), and legs (Figure 2g), while falcate scales belong to the dorsal side of the thorax (Figure 2h).

**Figure 3:**
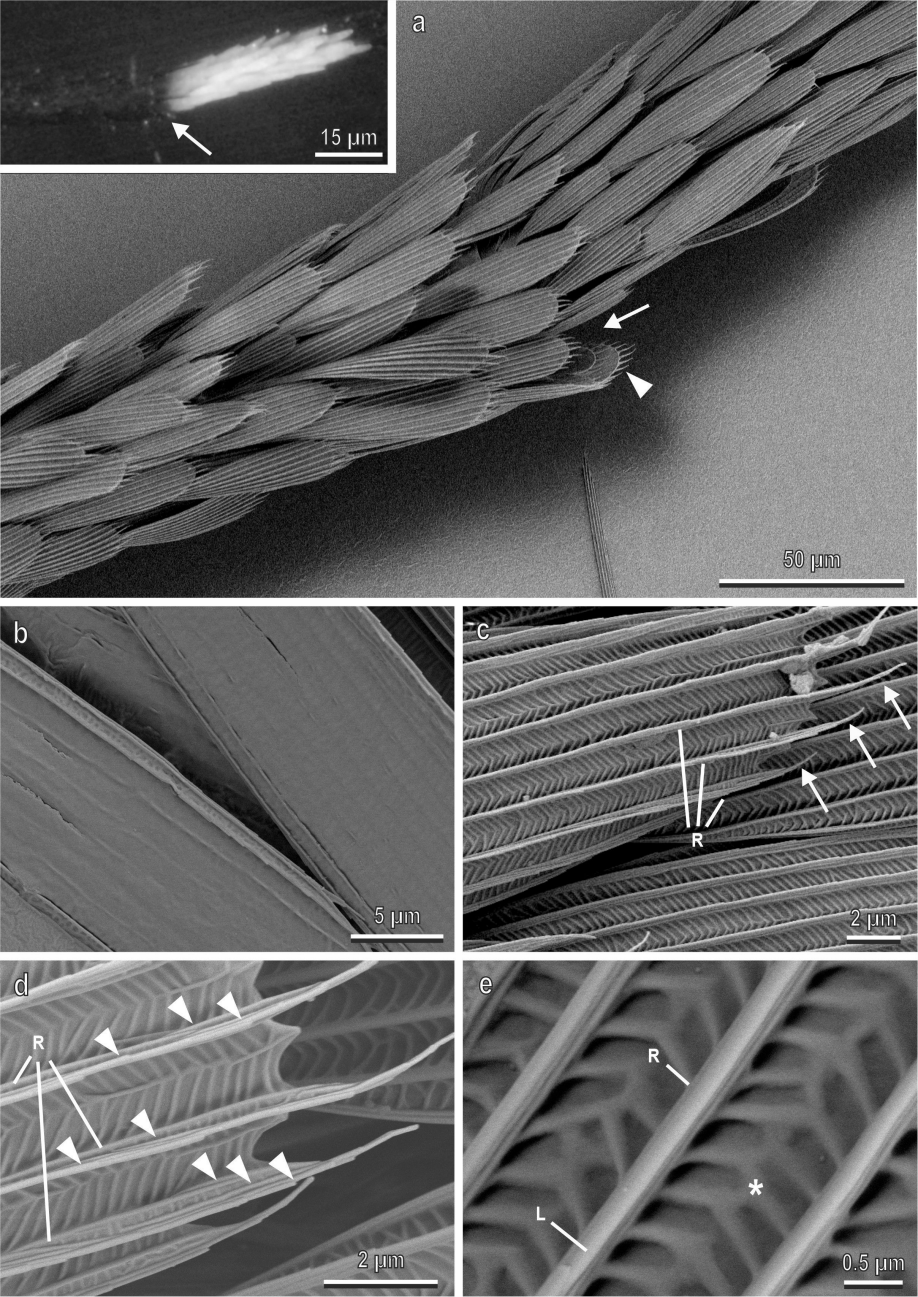
Tarsal black and white scales of *Aedes albopictus* (female) in a cryo-SEM. (a) Scales at the articulation (arrow) between the tarsal segments of the hindlegs, where the black scales are close to the white scales (in the inset the same image under stereomicroscope is shown). Note that both scales are spatulate, convex and show a rounded apex. Their size is variable, and some small scales are visible under wider scales (arrow head). (b) The lower surface of two scales without special micro- or nanostructures. (c) Upper surface of the scale with numerous longitudinal ridges (R) extending beyond the apical portion of the scale thus forming an apical fringe (arrows). (d) Detail of the longitudinal ridges (R) constituted of overlapping cuticular folds generating microribs (arrow heads) along the sides of the ridges. (e) Cuticle between longitudinal ridges (R) decorated with anisotropically situated nanostructures with a herringborne pattern (asterisk). L, lamella.

To analyse the micro- and nanostructures located on the scales of *Ae. albopictus* in detail, we focused on the tarsi of the hindlegs, which show particularly evident white patches. In particular, we examined the scales at the articulation between different tarsal segments of the hindlegs, where black scales are situated very close to white scales (Figure 3a and inset). Scales are spatulate, convex and show a rounded apex. Their size is variable, and some small scales are visible under wider scales (Figure 3a). The lower surface of a scale is flat, without specialized micro- and nanostructures (Figure 3b). The upper surface is covered by longitudinal ridges (Figure 3c–e). The distance between the ridges is 1.189 ± 0.03 µm (average ± SE) in the black scales and 1.283 ± 0.03 µm in the white scales. The cuticle between longitudinal ridges is decorated with anisotropically situated nanostructures with a herringbone pattern (Figure 3e). The ridges show overlapping lamellae from which fine folds or microribs run down the sides, along the sides of the ridges (Figure 3d). The longitudinal ridges extend beyond the apical portion of the scale, thus forming an apical fringe (Figure 3c).

**Figure 4:**
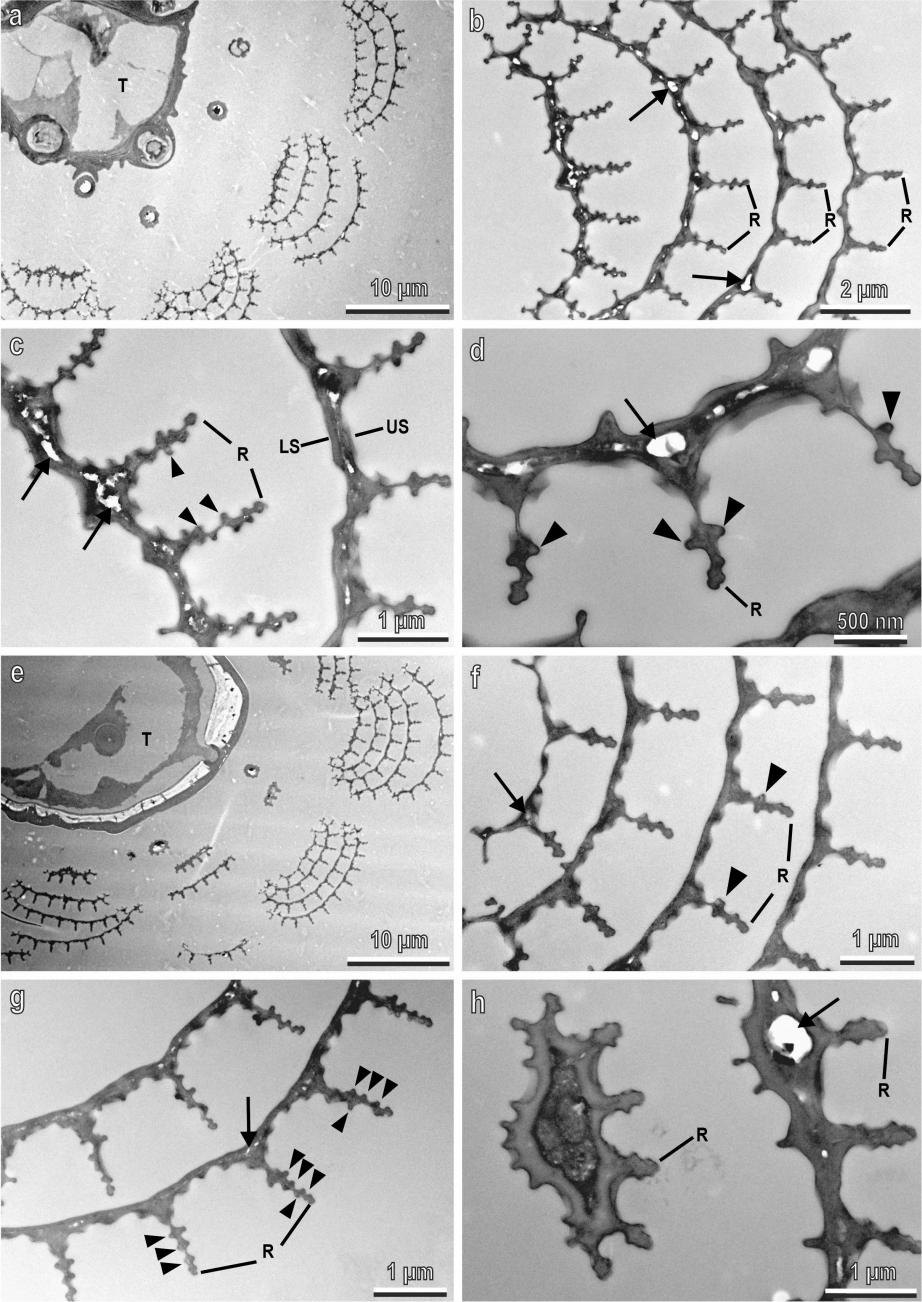
Tarsal black and white scales of *Aedes albopictus* female in a TEM. Cross sections of the tarsi in their white (a–d) and black (e–h) portions. In (a) and (e) note the series of overlapping four to five convex scales around the tarsus (T). The lower (LS) and the upper (US) surfaces are closely juxtaposed even if some empty spaces representing nanovoids, originating from the rest of epidermal cells, are occasionally visible between them (arrows). The cuticular microribs (arrow heads) along the ridges (R) appear in the cross section as small lateral globular bulges.

Some details of the nanostructures, characterising the tarsal scales of *Ae. albopictus,* have been clarified with the aid of TEM (Figure 4). Cross sections of the tarsi in their white (Figure 4a–d) or black (Figure 4e–h) portions reveal that series of four to five convex scales overlap each other (Figure 4a,b,e,f). The scales are very thin (about 0.2 µm thick). Their lower and the upper surfaces are closely juxtaposed, even if some empty spaces (nanovoids) are occasionally visible between them (Figure 4b–d,h). Such nanovoids originate from the rests of epidermal cells and appear in TEM as white or light grey areas inside the scales, together with electron-dense debris (Figure 4h). Their occurrence is higher at the bases of microribs, because the cuticle thickness is higher there (Figure 4h). Along the upper surface of the scale, the longitudinal ridges run orthogonal with respect to the herringbone pattern floor (Figure 4b,c,f,g). The cuticular microribs along the ridges appear in the cross section as small lateral globular bulges (Figure 4c,d,f,g). We did not observe any clear difference in the morphology of black and white scales in SEM and TEM, except for the small difference in the distance between the longitudinal ridges.

**Figure 5:**
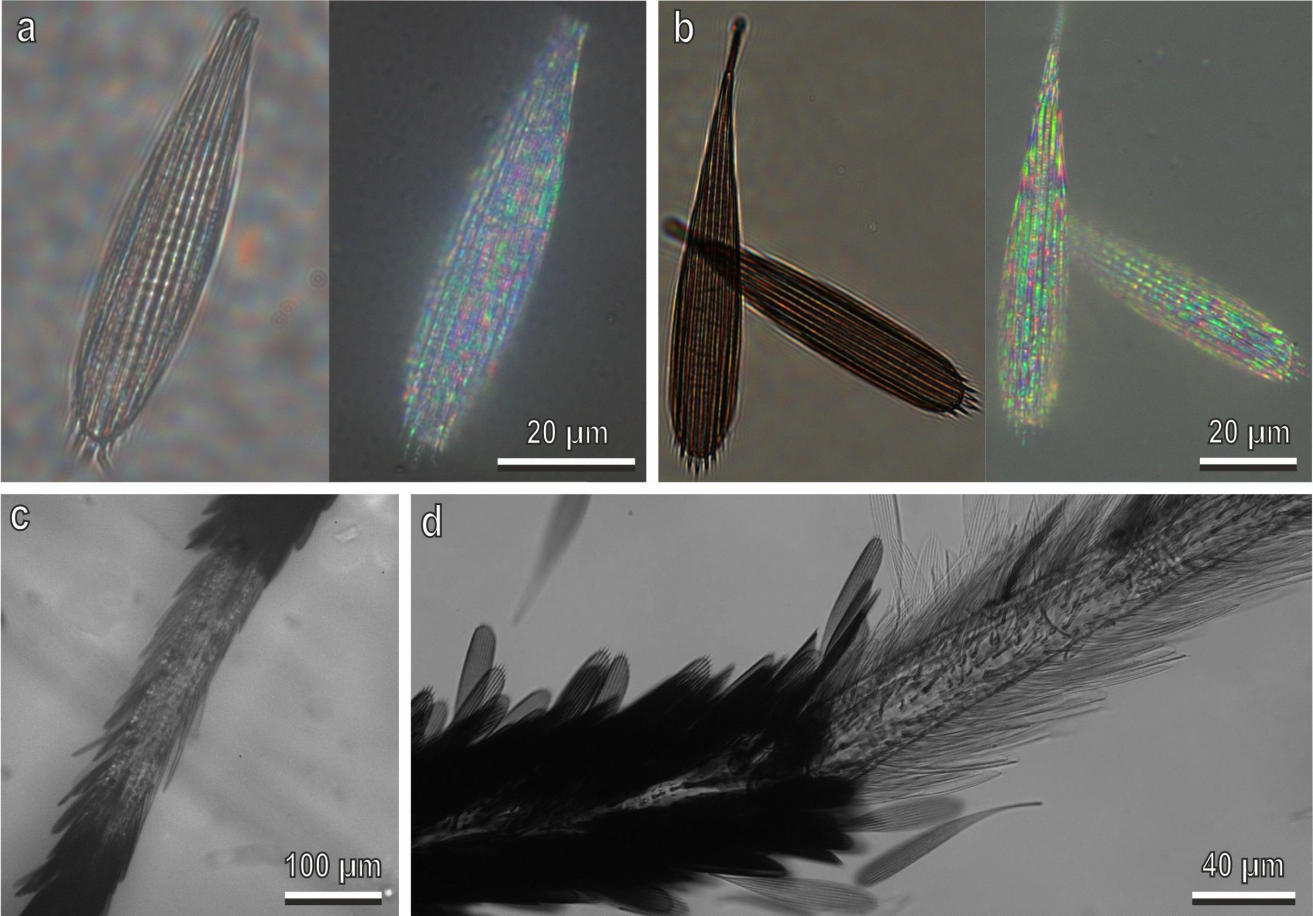
Tarsal black and white scales of *Aedes albopictus* female in a light microscope. (a) Dry white scales under transmitted (left) and reflected (right) light; (b) Dry black scales under transmitted (left) and reflected (right) light. (c) White and black scales immersed in oil under reflected light. Note that the white scales do not appear white, but rather transparent, while the black scales appear dark due to the presence of a certain amount of dark pigment in the cuticle. (d) White and black scales immersed in oil under transmitted light.

**Figure 6:**
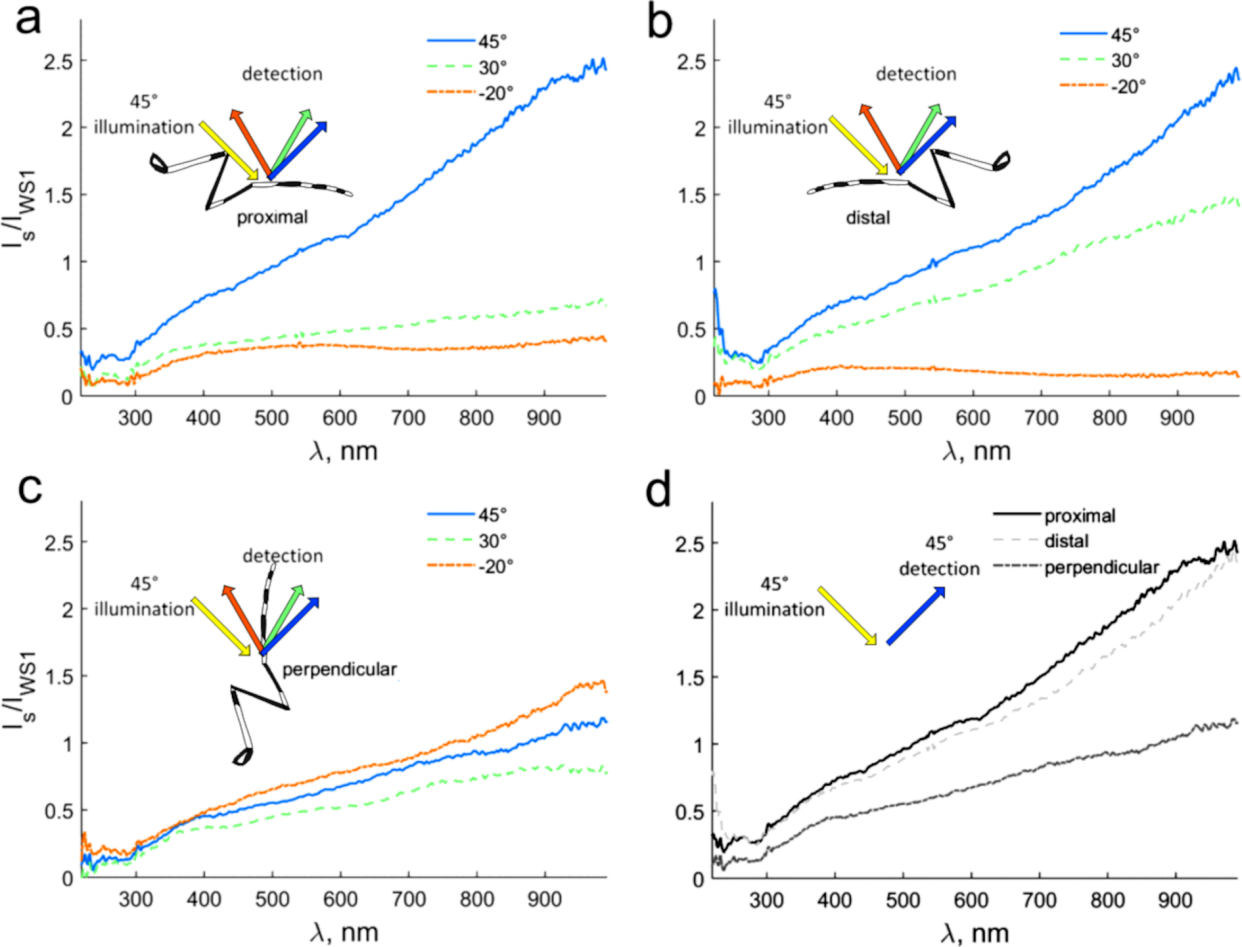
The light scattering spectra from a white area of the hindleg tarsus of *Aedes albopictus* male at different leg orientations. The white spot was illuminated at 45°. The light scattering was detected at 45°, 30°, and −20° (relative to the normal to the surface). Illumination was performed at different leg orientation relative to the illumination source: illumination was from the proximal direction (a), from the distal direction (b), and perpendicular to the tarsus main axis (c). Reflection at 45° for different leg orientations is summarized in (d).

The scales appear black and white only under reflected light at low magnification. In our observation of the tarsal scales of *Ae. aegypti* in a light microscope under transmitted and reflected light we could observe that in dry specimens under transmitted light white scales appear transparent and black scales appear dark (Figure 5a,b). Under reflected light, at high magnification, both white and black scales demonstrate a faint iridescent spotted pattern (Figure 5a,b). The colour of the pattern depends on the illumination direction in black scales, while in the white scales only the spot saturation changes at different illumination directions. Scales loose the whitish coloration when observed immersed in oil (with a refractive index closer to that of the cuticle material as to that of air) under reflected (Figure 5c) and transmitted (Figure 5d) light. White scales look transparent under these conditions, white black scales remain dark because of melanization. All reflection spectra are homogenous without evident peaks (Figure 6). The reflection intensity in the near-infrared spectral range (>900 nm) is about 2.5 times stronger than the WS1 reflection (!) and up to five times stronger (at 45° detection) than in the UV spectral range (<300 nm) (Figure 6). Pronounced specular reflection (at 45° detection) could be seen at proximal and distal illumination (Figure 6). Enhanced reflection at 30° detection and distal illumination (compared with proximal illumination) (Figure 6a,b) should be related to the tilt angle of the individual scales (Figure 3a). There is also relative strong light scattering on the thin scale surface structure (Figure 3c–e; Figure 4), which amounts for even backwards reflection (proximal illumination, −20° detection) of about 1/3 of the WS1 standard reflection (at 45° illumination/detection). From geometrical considerations, it is clear that backwards reflection (−20° detection) is the strongest and reflection at 30° is the weakest at perpendicular illumination. In UV spectral range, the light scattering has no specific trends (corresponding to, e.g., Rayleigh scattering), though absorption by molecules with aromatic groups might modulate the reflection. There are no pigments in the white scales (Figure 5d), so interference on the single scales structure is responsible for the reduced visible and UV (compared to near-IR) reflection.

**Figure 7:**
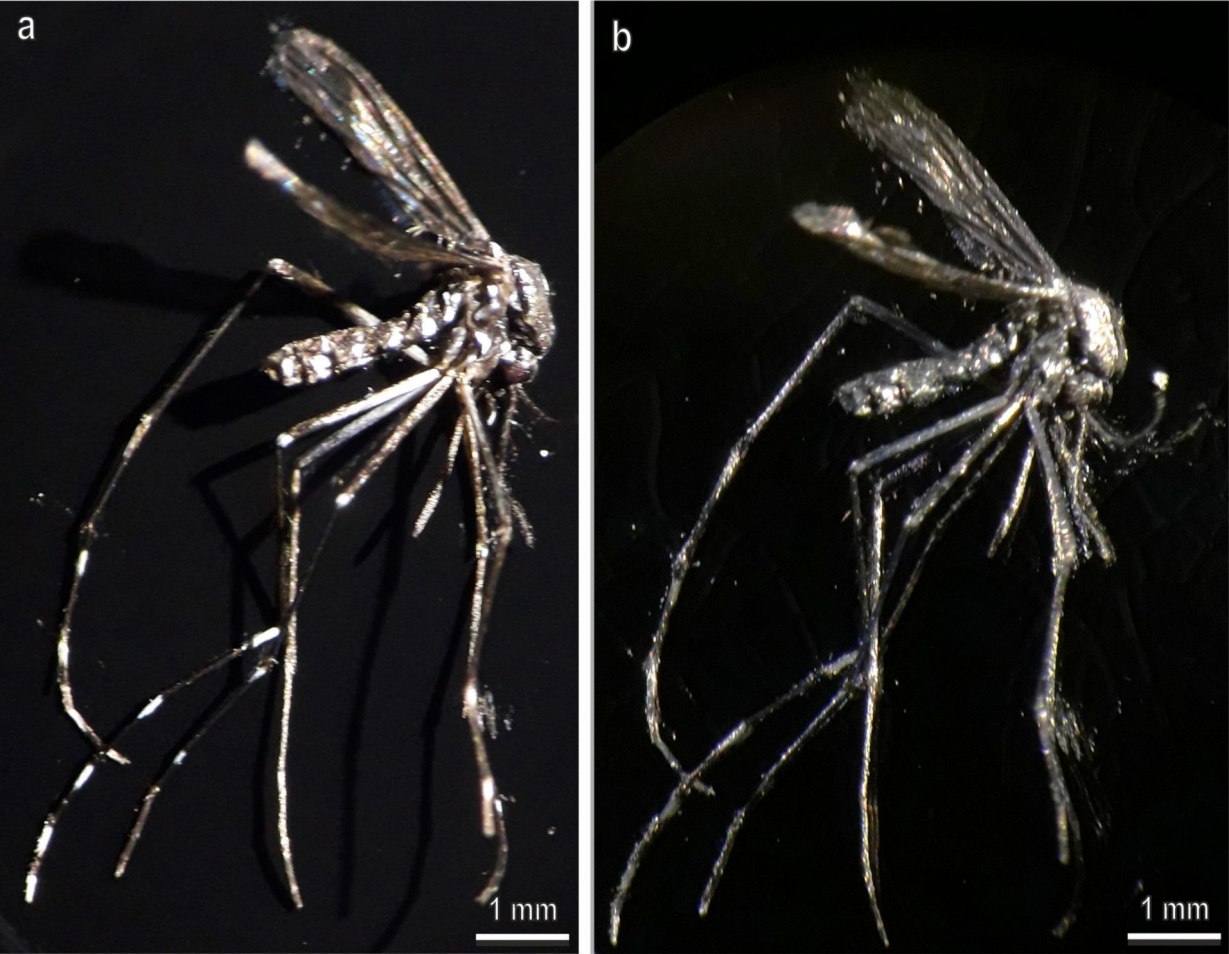
*Aedes albopictus* before (a) and after (b) Au/Pd sputter coating (10 nm film thickness). Note that in (b) the thin Au/Pd film screens the pigment inside the scales and eliminates the visual difference between white and black scales originally visible in the intact animal (a).

Sputter coating with Au/Pd (10 nm thickness) of the tarsal scales of *Ae. albopictus* (Figure 7) almost eliminates the visual difference between white and black scales. Since the scale surface is optically smooth, the metal-coated scales demonstrated specular reflection. A thin Au/Pd film on the scale surface effectively screens the pigment presented inside the scales and traps the light so that the white as well as the black appearance of the scales disappears.

**Figure 8:**
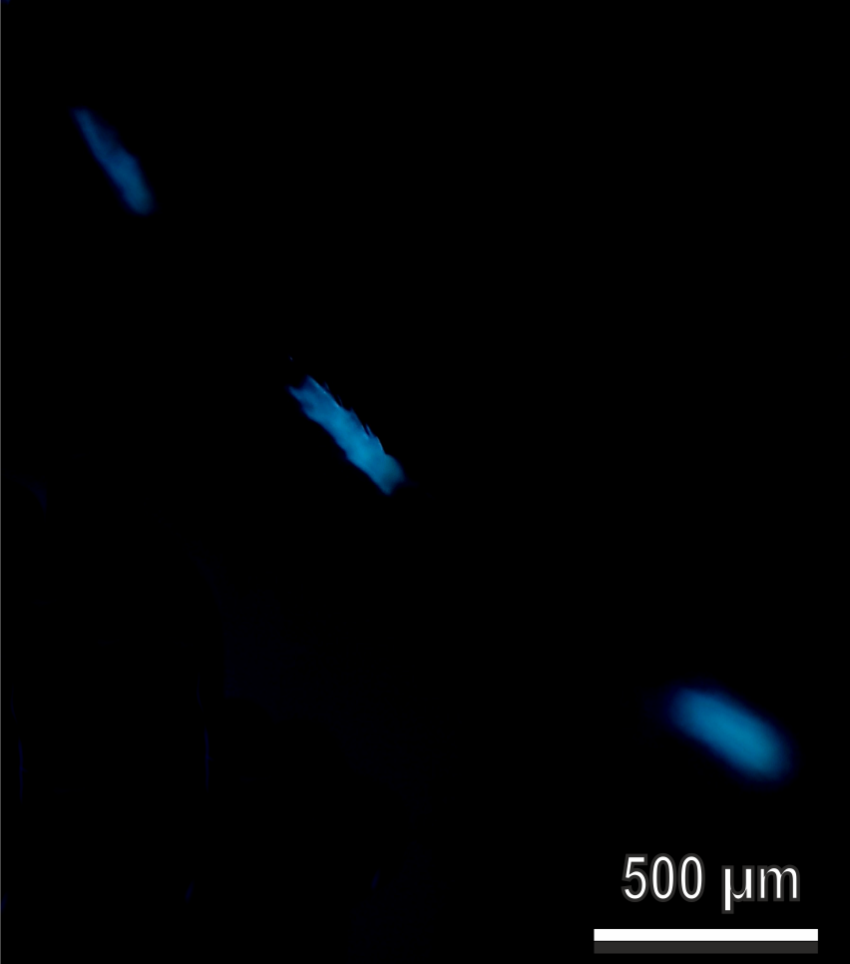
Tarsal black and white scales of *Aedes albopictus* (female) in a fluorescence microscope. Tarsi observed with an excitation filter 365 nm, chromatic beam splitter FT 395 nm, emission 397 nm. Note the blue autofluorescence of the white areas of the tarsi.

Observations of the legs of dry adults using a fluorescence microscope revealed that the white areas on the tarsi show UV-induced fluorescence (Figure 8). The excitation occurs at 365 nm (UV light) and the emission from 397 nm (blue light).

## Discussion

### Multifunctional role of the scales

The present investigation reveals that the white bright scales on the body of *Ae. albopictus* are constituted of transparent cuticle, and their micro- and nanostructures can generate structural white. These results suggest a multifunctional role of the mosquito scales which, in addition to a superhydrophobic function [11–13], produce structural colours similar to those of butterfly scales. The involvement of these structures in the origin of structural white is clearly demonstrated by our optical microscopy observations. Indeed, when we observed the tarsal scales under transmitted and reflected light, we could observe white bright scales only in dry specimens under reflected light, while the white scales observed under transmitted light appear transparent. Moreover, the white scales immersed in oil with a refractive index close that of chitin do not appear white, but rather transparent, while the black scales appear dark due to the presence of a certain amount of a dark pigment melanin in the cuticle. This means that neither pigments nor inclusions with strongly different refractive index (e.g., voids) are responsible for white appearance of the scales.

Structural whiteness requires scattering processes for all visible wavelengths [19]. Two kinds of structural whiteness can be recognised, namely diffuse whiteness, which is angle-independent and metallic silver, which is angle-dependent. Diffuse whiteness produces matt white and is due to the multiple scattering of light within a randomly structured medium, such as the three-dimensional photonic solid in the scales of *Cyphochilus* spp. beetles [20]. An example of structural white in Diptera has been recently described in the white patches located on the thorax of *Bactrocera oleae* (Rossi) (Diptera: Tephritidae) [21]. In this fly, the structural white is due to modified air sacs under transparent cuticle and is independent on the diffuse angle. This structure shows internal arborisations with beads in an empty space, constituting a three-dimensional photonic solid responsible for light scattering. Metallic silver produces a bright, shining white caused by a variation of the reflected light intensity under different incident or viewing angles, such as in the scales on the ventral side of the wings of the butterfly *Curetis acuta* Moore (Lepidoptera: Lycaenidae) [22] or in the *Ae. albopictus* scales described here. Indeed, the structural white produced by the micro- and nanostructures of the scales on the tarsi of *Ae. albopictus* appears bright and is angle-dependent since the reflected light changes according to the angle detection and according to the tarsus orientation (from the proximal direction, from the distal direction, and perpendicular to the tarsus main axis).

### Optical effects on the mosquito leg scale system

The optical appearance of the scale system of *Ae. albopictus* has a complex nature and can be explained by the combination of several effects, which contribute to different extent: (1) the cylindrical shape of the leg, which may alter scattering depending on the illumination/observation configuration; (2) light scattering, which might be subdivided in (a) Rayleigh scattering on nanostructures, (b) scattering on partially regular micro- and nanostructures, and (c) thin film interference scales; (3) selective absorption by various molecules present in the cuticle; and (4) collective effects of the overlapping scales. The whole structural hierarchy determines the reflection properties of the white scales, but different micro- and nanostructures are presumably involved in the light scattering at different illumination/detection conditions. Below, we provide a detailed discussion of these effects and their contribution to the measured spectra.

#### Cylindrical shape of the leg

The cylindrical shape of the leg in combination with the structural white makes the white scales well visible, independent on the observation direction in the plane perpendicular to the legs (Figure 6c). The reason for this is that in the plane crossing the cylinder perpendicular to the cylinder axis, a point on the cylinder surface exists where the incident and reflected angles (from the light source and to the detector, respectively) are equal.

#### Rayleigh scattering

The contribution of the Rayleigh scattering to the white appearance is almost negligible. The scales look slightly bluish under a light microscope, but there is no evidence for a λ^−4^-dependence (typical for Rayleigh scattering) in the reflection spectra that increases with increasing wavelength (Figure 6). Besides, the scales are transparent when they are immersed into oil. This means, that there are no or just a few intracuticular nanostructures whose refractive index differs from that of the cuticle itself. In the TEM images, only a small number of nanovoids could be found (Figure 4).

#### Scattering on micro- and nanostructures

Multiple refraction and reflection on the ridges and microribs, as well as reflection from the upper surface of the scales between ridges (and the corresponding herringbone surface pattern) are mainly responsible for the white appearance of the scales. Coating of scales with a thin Au/Pd layer eliminates the white (and also the black) appearance and demonstrates the importance of multiple reflections for the appearance of the white scales (Figure 6c).

#### Thin film interference

In the microscopic areas on the scales with relatively constant cuticle thickness, the effect of interference in thin films can be observed, which modulates the reflection spectra. Its contribution is about 11% (considering the refractive index of chitin of 1.56 and an illumination angle of 45°), and it is visible as faint colours on the white background in microscopic observations (Figure 5a). A similar but weaker interference is present in the black scales (Figure 5b) because of the partial light absorption by melanin.

#### Light absorption

Some substances absorbing UV light are present even in the white scales. They attenuate the reflection in the UV–visible, but not in the red–IR, spectral range. Multiple refractions and reflections enhance this effect.

#### Collective effects of the overlapping scales

Finally, the fact that the scales overlap each other (Figure 5d) may lead to an increase of both reflection and scattering. Thus, the specular reflection from four overlapping chitin scales at 45° should be three times stronger than the reflection from a single scale. The variation in the scale orientation at the level of one scale and between neighbouring scales is responsible for the strong small-angle scattering and for the absence of the prominent specular reflection. The scales are tilted with respect to the surface and directed distally (Figure 5d). Highly diffuse scattering might be the only reason for the strong reflection at 30° and distal illumination compared to proximal illumination (Figure 6a,b).

Therefore, the main contribution to the white coloration is provided by non-coherent light scattering (diffuse scattering) on hierarchical randomized multiscale 3D structures (Figure 4a–d, Figure 3d). The coherent contribution (interference), which was estimated to be around 10%, appears due to structure regularity.

### Functional significance of the mosquito leg scale system

Structural colors producing matte white and bright silver brilliancy can be readily distinguished and can be involved in inter- and intraspecific visual communication including recognition and mating [23–24]. Our data on *Ae. albopictus* scales and the above discussion show that the main contribution to the white appearance of the scale system comes from the multiple refraction and reflection on the ridges and microribs. Interestingly the white scales of *Ae. albopictus* show UV-induced blue autofluorescence as it has been demonstrated by [24] and confirmed here, exactly as it happens in the white patches of the olive fruit fly *B. oleae* [21]. The biological role of white patches is not known, neither in the olive fruit fly *B. oleae* nor in the mosquitoes of the genus *Aedes*. However, we can hypothesize a role in mate recognition in *Ae. albopictus*. The use of visual cues in mate recognition has been described in other Diptera, such as the two predatory fly species *Lispe consanguinea* Loew (Diptera: Muscidae) and *L. tentaculata* DeGeer (Diptera: Muscidae), which use visual cues from reflective concave silvery scales on the head face during mating [25]. It is well known that in mating swarms, mosquitoes mainly use acoustic signals created by conspecific wingbeats to locate and respond to one another through harmonic convergence [26]. Yet, visual trap components can be important, especially in diurnally active mosquitoes, such as the yellow fever mosquito *Ae. aegypti* (and the tiger mosquito *Ae. albopictus*). Indeed, it has been previously demonstrated that the use of visual cues can improve the attractiveness of sound-baited traps [27–28]. The mechanisms underlying swarm formation and long-range detection of females by males remain largely unexplored in mosquitoes of the genus *Aedes,* but an investigation [29] demonstrated that swarm formation and mate recognition in *Ae. aegypti* are mediated, in part, by visual cues represented by wingbeat light flashes. Such flashes had no signal function for crepuscular house mosquitoes, such as *Culex pipiens* L. (Diptera: Culicidae) [29].

Another possible role of the black and white coloration of the *Aedes* genus could be related to defensive behaviour. The influence of prey coloration of animals living in groups on the “confusion effect” towards predators is a field still largely unexplored. However, there is some evidence in “human predators” that motion dazzle camouflage could enhance the confusion effect [30–32]. In this context, fast moving black-and-white mosquitoes reflecting light that changes owing to the angle-dependent structural white produced by the scales could create a visual effect causing confusion in predators and disrupting perception. Further studies are necessary to test this hypothesis.

In conclusion, the present research analysed in detail the ultrastructure of the scales producing bright white areas alternating with dark areas on the tarsi of the mosquito *Ae. albopictus.* We could identify micro- and nanostructures responsible for the production of an angle-dependent structural white, confirmed by reflection spectra measurements of the white areas. We believe that to deepen the knowledge on the nanostructures producing structural colours in mosquitoes of the genus *Aedes,* is important not only to widen our knowledge on the origin, variability, and function of structural colours in insects, but also to better understand the nature of the cues potentially driving the behaviour of these medically and economically significant insects. This knowledge will allow for the development of more effective traps to collect, monitor, and control these dangerous insect species.

## Experimental

### Insects

Single alive females of *Ae. albopictus* were collected in Perugia, Italy in vials using human beings as attractants. Insects were placed in a net cage (300 mm × 300 mm × 300 mm) and transferred to the laboratory. In the laboratory, some specimens were immediately fixed for TEM investigations, while some specimens were frozen at −20 °C for further use. For the terminology regarding the morphological description of mosquito scales we used as reference [16].

### Light microscopy

Tarsal black and white scales of *Ae. albopictus* were observed using light microscopy with transmitted and reflected light in an inverted bright-field microscope ZEISS Axio Observer (Carl Zeiss Microscopy GmbH). Dry specimens and specimens immersed in oil (immersion oil 518, Carl Zeiss, Oberkochen, Germany) were observed. Sputter-coated samples (see SEM procedure) were observed using a stereomicroscope under reflected light.

### Fluorescence microscopy

Observations of the legs of dry adults were performed using a fluorescence microscope Zeiss Axiophot (Zeiss, Jena, Germany) with an excitation filter 365 nm, chromatic beam splitter FT 395 nm, emission 397 nm, with an excitation filter 450–490 nm, chromatic beam splitter FT 510 nm, emission 520 nm, and with an excitation filter 546 nm, chromatic beam splitter FT 580 nm, emission 590 nm. Insect legs were removed from the insects and placed on a microscope slide for observations.

### Scanning electron microscopy and cryo-SEM

Dry insects were observed in a scanning electron microscope Hitachi S-4800 (Hitachi High-Technologies Corp., Tokyo, Japan) at 3 kV acceleration voltage. Specimens were sputter-coated with gold/palladium (10 nm layer thickness).

In a similar manner to a procedure from [33], for cryo-SEM, insect samples were either glued with Tissue-Tek^®^ O.C.T.TM Compound (Sakura^®^ Finetek Europe B.V., Zoeterwoude, Netherlands) to metal holders or mechanically gripped in a small vice on holders and then frozen in a cryo-stage preparation chamber at −140 °C (Gatan ALTO2500 cryo-preparation system, Gatan Inc., Abingdon, UK). Frozen samples, either whole or fractured with a cold metal fracture knife in the cryo-stage preparation chamber, were sputter-coated with gold/palladium (6 nm thickness) and examined under freeze conditions (−120 °C) in the SEM Hitachi S-4800 at 3 kV acceleration voltage. Sputter-coated samples were also used to demonstrate the modulation of the light reflection properties of both black and white scales after introduction of a metal layer on top of the scales.

### Transmission electron microscopy

In a similar manner to a procedure from [21], the tarsi and tibiae of *Ae. albopictus* were dissected from anaesthetized insects and fixed for 3 h in 2.5% glutaraldehyde in cacodylate buffer (Electron Microscopy Sciences, Hatfield, England), pH 7.2. The fixed legs were repeatedly rinsed in sodium cacodylate buffer and post-fixed for 1 h at 4 °C in 1% osmium tetroxide in sodium cacodylate buffer (Electron Microscopy Sciences). The samples were then repeatedly washed in the same buffer, dehydrated in ascending ethanol concentrations, and finally embedded in an Epon-Araldite resin mixture (Sigma-Aldrich). Afterwards, ultrathin sections were cut using a Leica EM UC6 ultramicrotome (Leica Microsystem GmbH, Wetzlar, Germany), collected on formvar-coated copper grids, and examined using a TEM Philips EM 208 (Philips, Eindhoven, Netherlands).

### Reflectance spectra measurements

The light scattering spectra from the white areas on the proximal parts of the hind leg tarsus of *Ae. albopictus* male were measured at different orientations of the light source relative to the leg and at different detection angles. For this purpose, the observation area was illuminated at 45° with a light source (DH-2000-BAL, Ocean Optics Inc, Dunedin, Florida, USA) through an optical fiber (200 μm diameter) equipped with a one-lens condenser, which was placed 22 mm away from the sample. The illumination spot was one millimetre in diameter. The illuminated area was calculated based on the microscopy image of the leg (similar to Figure 1f). The mosquito hind leg was fixed using double-sided adhesive tape (Tesa^®^, Hamburg, Germany) so that the tarsus was parallel to the horizontal plane with the dorsal side up. Measurements were performed for three different orientations of the tarsus relative to the light source, namely illumination along the tarsus main axis proximally and distally, and perpendicular to the tarsus main axis.

The light scattering was relatively homogeneous. Therefore, just three different angles were selected to characterize the reflection properties of the white areas. Reflection spectra at angle of incidence (45°, from the normal to the surface), small-angle scattering (30°), and the backscattering (−20°) were measured. The scattered light was collected by another condenser, which was placed 46 mm away from the sample and was mounted on an optical fiber (200 μm diameter), similarly to [18]. The optical fiber was connected to a spectrometer (Ocean Optics Inc, Dunedin, Florida, USA). The spectra were recorded with the software Spectral Suite (Ocean Optics Inc, Dunedin, Florida, USA). Further spectral processing was performed in Matlab 7.10 (The MathWorks, Natick, NA, USA) and includes dark noise subtraction and smoothing with a Savitzky–Golay filter of fourth order with 12 nm window. All spectra were normalized to the WS1 standard (Ocean Insight, Ostfildern, Germany) reflection spectrum (illumination and observation at 45°). No correction on the observation angle was performed.

## References

[1] Cuthill I C, Allen W L, Arbuckle K, Caspers B, Chaplin G, Hauber M E, Hill G E, Jablonski N G, Jiggins C D, Kelber A (2017). Science.

[2] Shamim G, Ranjan S K, Pandey D M, Ramani R (2014). Eur J Entomol.

[3] Burg S L, Parnell A J (2018). J Phys: Condens Matter.

[4] Kinoshita S, Yoshioka S, Miyazaki J (2008). Rep Prog Phys.

[5] Ghiradella H (1989). J Morphol.

[6] Vukusic P, Sambles J R, Lawrence C R, Wootton R J (1999). Proc R Soc London, Ser B.

[7] Berthier S, Charron E, Da Silva A (2003). Opt Commun.

[8] Theobald F V (1901). J Trop Med.

[9] Theobald F V (1905). Diptera. Fam. Culicidae. Genera Insectorum.

[10] Khalaf K T, Khalaf R K (1984). Fla Entomol.

[11] Wu C W, Kong X Q, Wu D (2007). Phys Rev E.

[12] Kong X Q, Liu J L, Zhang W J, Qu Y D (2015). AIP Adv.

[13] Iikura H, Takizawa H, Ozawa S, Nakagawa T, Matsui Y, Nambu H (2020). Sci Rep.

[14] Wilkerson R C, Peyton E L (1990). J Med Entomol.

[15] Westphal-Ferreira B, Navarro-Silva M A (2017). Rev Bras Entomol.

[16] Ghirardella H (2010). Adv Insect Physiol.

[17] Seago A E, Brady P, Vigneron J-P, Schultz T D (2009). J R Soc, Interface.

[18] Hawley W (1988). J Am Mosq Control Assoc.

[19] Stavenga D G (2014). Mater Today: Proc.

[20] Vukusic P, Hallam B, Noyes J (2007). Science.

[21] Rebora M, Salerno G, Piersanti S, Kovalev A, Gorb S (2021). Commun Biol.

[22] Liu X, Wang D, Yang Z, Zhou H, Zhao Q, Fan T (2019). Adv Opt Mater.

[23] Ge D, Wu G, Yang L, Kim H-N, Hallwachs W, Burns J M, Janzen D H, Yang S (2017). Proc Natl Acad Sci U S A.

[24] Croce A C, Scolari F (2022). Molecules.

[25] Frantsevich L, Gorb S (2006). Arch Insect Biochem Physiol.

[26] Steele C H, McDermott E G (2022). Ann Entomol Soc Am.

[27] Balestrino F, Iyaloo D P, Elahee K B, Bheecarry A, Campedelli F, Carrieri M, Bellini R (2016). Acta Trop.

[28] Jakhete S S, Allan S A, Mankin R W (2017). J Med Entomol.

[29] Ko E, Blake A J, Lier C, Takács S, Gries G (2021). bioRxiv.

[30] Hogan B G, Cuthill I C, Scott-Samuel N E (2016). Behav Ecol.

[31] Hogan B G, Cuthill I C, Scott-Samuel N E (2017). Anim Behav.

[32] Hogan B G, Scott-Samuel N E, Cuthill I C (2016). R Soc Open Sci.

[33] Gorb E V, Kozeretska I A, Gorb S N (2022). Beilstein J Nanotechnol.

